# Chronic Morphine Alters the Presynaptic Protein Profile: Identification of Novel Molecular Targets Using Proteomics and Network Analysis

**DOI:** 10.1371/journal.pone.0025535

**Published:** 2011-10-17

**Authors:** Noura S. Abul-Husn, Suresh P. Annangudi, Avi Ma'ayan, Dinah L. Ramos-Ortolaza, Steven D. Stockton, Ivone Gomes, Jonathan V. Sweedler, Lakshmi A. Devi

**Affiliations:** 1 Department of Pharmacology and Systems Therapeutics, Mount Sinai School of Medicine, New York, New York, United States of America; 2 Department of Chemistry, Beckman Institute, University of Illinois at Urban-Champaign, Urbana, Illinois, United States of America; Semmelweis University, Hungary

## Abstract

Opiates produce significant and persistent changes in synaptic transmission; knowledge of the proteins involved in these changes may help to understand the molecular mechanisms underlying opiate dependence. Using an integrated quantitative proteomics and systems biology approach, we explored changes in the presynaptic protein profile following a paradigm of chronic morphine administration that leads to the development of dependence. For this, we isolated presynaptic fractions from the striata of rats treated with saline or escalating doses of morphine, and analyzed the proteins in these fractions using differential isotopic labeling. We identified 30 proteins that were significantly altered by morphine and integrated them into a protein-protein interaction (PPI) network representing potential morphine-regulated protein complexes. Graph theory-based analysis of this network revealed clusters of densely connected and functionally related morphine-regulated clusters of proteins. One of the clusters contained molecular chaperones thought to be involved in regulation of neurotransmission. Within this cluster, cysteine-string protein (CSP) and the heat shock protein Hsc70 were downregulated by morphine. Interestingly, Hsp90, a heat shock protein that normally interacts with CSP and Hsc70, was upregulated by morphine. Moreover, treatment with the selective Hsp90 inhibitor, geldanamycin, decreased the somatic signs of naloxone-precipitated morphine withdrawal, suggesting that Hsp90 upregulation at the presynapse plays a role in the expression of morphine dependence. Thus, integration of proteomics, network analysis, and behavioral studies has provided a greater understanding of morphine-induced alterations in synaptic composition, and identified a potential novel therapeutic target for opiate dependence.

## Introduction

Repeated exposure to opiates, such as morphine, produces significant and persistent changes in synaptic transmission and plasticity that may contribute to altered behaviors associated with addiction, dependence and withdrawal. While the molecular and cellular mechanisms underlying these long-lasting changes are not fully understood, substantial evidence shows that opiates play a critical role in the modulation of neurotransmitter release, particularly in the mesolimbic dopaminergic system. Chronic morphine exposure increases dopamine signaling in structures of this system [1]–[6], including the ventral striatum, involved in reward [7]; and the dorsal striatum, involved in craving and relapse [8]. Since reward, craving and relapse contribute to the development and maintenance of opiate addiction, it is likely that presynaptic proteins involved in the regulation of neurotransmitter release in the striatum participate in the synaptic adaptations mediating opiate addiction, dependence and withdrawal.

Given the importance of presynaptic neurotransmitter release in drug addiction, we undertook a quantitative subcellular proteomic analysis to investigate the effects of morphine on striatal presynaptic protein levels. Proteomics serves as a powerful tool to reveal changes in protein abundance in response to drug administration [9]. While many studies have described proteome changes in different brain regions [10]–[16] and cell culture preparations [17]–[18] following chronic morphine administration, few have examined morphine-induced changes in the synaptic subproteome and none have used network analysis methods to predict novel protein complexes and signaling pathways altered by morphine.

Here we used an integrated proteomics, graph theory-inspired network analysis, and behavioral approach to elucidate the presynaptic molecular events induced by repeated morphine administration. This has enabled a greater understanding of morphine-induced alterations in synaptic composition, and has allowed the identification of potential therapeutic targets for opiate dependence and addiction.

## Results

### Protein identification and quantification

**Figure 1:**
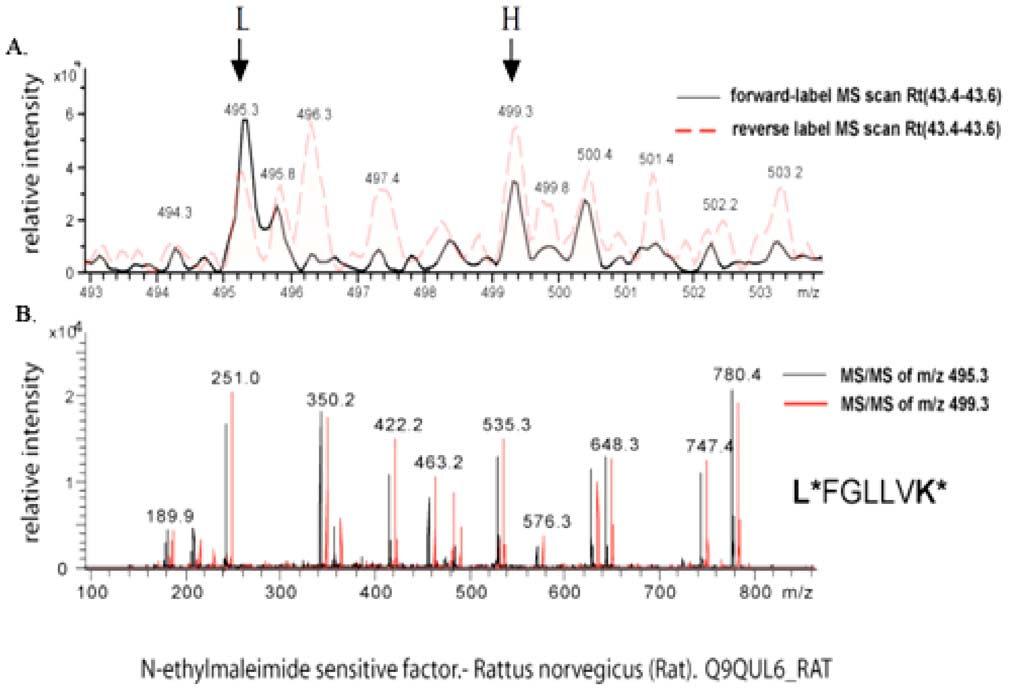
Representative spectra of NSF following differential isotopic labeling and LC-MS/MS. Protein extracts from striatal PRE fractions of saline- and morphine-treated rats were labeled either with succinic anhydride (light) or deuterated succinic anhydride (heavy) and analyzed by LC-MS/MS. (**A**) Combined MS scans showing the relative abundance of the peak pair with mass/charge (m/z) ratios 495.3 and 499.3 (indicated by the arrows). The morphine/saline ratio with forward labeling (saline = light, morphine = heavy; indicated in black) was ∼0.55. This ratio was reversed (morphine/saline ratio ∼1.6) with reverse labeling (saline = heavy (H); morphine = light (L); indicated in red). (**B**) Overlaid tandem MS of precursor ions m/z 495.3 (black) and m/z 499.3 (red) corresponding to the tryptic peptide L*FGLLK* with labels present on N-terminal amine and C-terminal lysine (indicated by the *). Mascot database search showed that the tryptic peptide was derived from N-ethylmaleimide sensitive factor (NSF).

**Figure 2:**
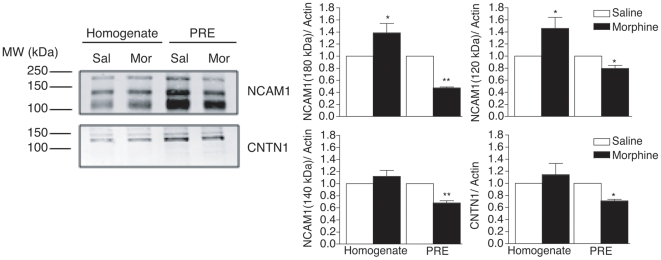
Validation of results from quantitative proteomics using Western blot analysis. The levels of NCAM and contactin 1 were decreased in the striatal PRE fraction of morphine-treated animals, but not in the total homogenate. The decrease in presynaptic NCAM was observed for the 180-, 140-, and 120-kDa isoforms of the protein.

To identify and quantify proteins regulated by morphine, presynaptic (PRE) proteins from saline- and morphine-treated rats were subjected to differential isotopic labeling and LC-MS/MS analysis. Five experiments were performed, using forward (saline = light, morphine = heavy) and reverse (saline = heavy, morphine = light) labeling (Table S1). A representative spectrum showing a decrease in NSF, a candidate protein, upon forward and reverse labeling is shown in Fig. 1A. Analysis of the MS/MS spectra led to the identification of 175 proteins (Table S2), 143 of which were quantified by determining the peak intensity of the labeled peptides (Fig. 1B). Only 30 of these proteins were robustly and consistently altered by morphine treatment; the majority of which were downregulated (Table 1). We confirmed the results from quantitative proteomic studies using Western blotting by verifying the decrease in some of these proteins from a separate set of saline- and morphine-treated animals (Fig. 2). The 30 proteins, included in a list designated as the “seed list”, belong to the following groups: vesicle trafficking (NSF, syntaxin binding protein 1); signaling (β_1_, β_2_, β_3_, and α_olf_ subunits of heterotrimeric G proteins), cytoskeleton-associated (septin 7, tubulin beta chain 7), chaperone (heat shock cognate 71 kDa or Hsc70), and cell adhesion (contactin 1, NCAM1). Several of these proteins have established roles in synaptic plasticity, while others have been reported to be altered in different paradigms of morphine treatment [10], [19], [20].

**Table 1:** Seed list of 30 proteins from the striatal PRE fraction that were altered by morphine treatment.

Gene Name	Protein Name	UniProt Acc. #
***Downregulated (23)***		
Vesicle Trafficking		
NSF	N-ethylmaleimide sensitive factor	Q9QUL6
AP2A2	AP-2 complex subunit alpha-2	P18484
STXBP1	Syntaxin-binding protein 1 (Unc-18 homolog)	P61765
Signaling		
GNAL	GTP-binding protein Golf alpha subunit	Q80WZ0
GNB1	Guanine nucleotide-binding protein G(I)/G(S)/G(T) subunit beta 1	P54311
GNB2	Guanine nucleotide-binding protein G(I)/G(S)/G(T) subunit beta 2	P54313
GNB3	Guanine nucleotide-binding protein G(I)/G(S)/G(T) subunit beta 3	P52287
YWHAZ	14-3-3 protein zeta/delta	P63102
Cytoskeleton-associated		
SEPT3	G-septin gamma	Q9R245
SEPT7	Septin 7	Q9WVC0
TUBB2B	Tubulin beta chain 15	Q3KRE8
Cell Adhesion		
CNTN1	Contactin 1	Q63198
NCAM1	Neural cell adhesion molecule 1, 140 kDa isoform	P13596
OPCML	Opioid-binding protein/cell adhesion molecule precursor (OBCAM)	P32736
Chaperone		
HSPA5	78 kDa glucose-regulated protein precursor (GRP 78)	P06761
HSPA8	Heat shock cognate 71 kDa protein	P63018
TCP1	T-complex protein 1 subunit alpha	P28480
Mitochondrial-related		
CS	Citrate synthase, mitochondrial precursor	Q8VHF5
DLD	Dihydrolipoyl dehydrogenase	Q6P6R2
HK1	Chain A, Rat Brain Hexokinase Type I Complex With Glucose And Inhibitor Glucose-6-Phosphate	P05708
HK2	Hexokinase 2	P27881
VDAC2	Voltage-dependent anion-selective channel protein 2	P81155
VDAC3	Voltage-dependent anion-selective channel protein 3	Q9R1Z0
***Upregulated (7)***		
Mitochondrial-related		
ATP5C1	ATP synthase gamma chain, mitochondrial	P35435
ATP5B	ATP synthase subunit beta, mitochondrial precursor	P10719
COX4I1	Cytochrome c oxidase subunit 4 isoform 1, mitochondrial precursor	P10888
COX5A	Cytochrome c oxidase subunit Va	P11240
COX5B	Cytochrome c oxidase subunit Vb	P12075
Kinase		
CDC42BPA	Serine/threonine-protein kinase MRCK alpha	O54874
Other		
HBB	Beta-globin	Q6PDU6

Proteins with morphine/saline ratios of at least 0.5 standard deviations from the mean and that showed consistent changes in at least 2 of the experiments were selected. The numbers in parentheses indicate the number of proteins that were downregulated or upregulated.

### Network Analysis: Integration of Proteomics Data into a PPI Network

**Figure 3:**
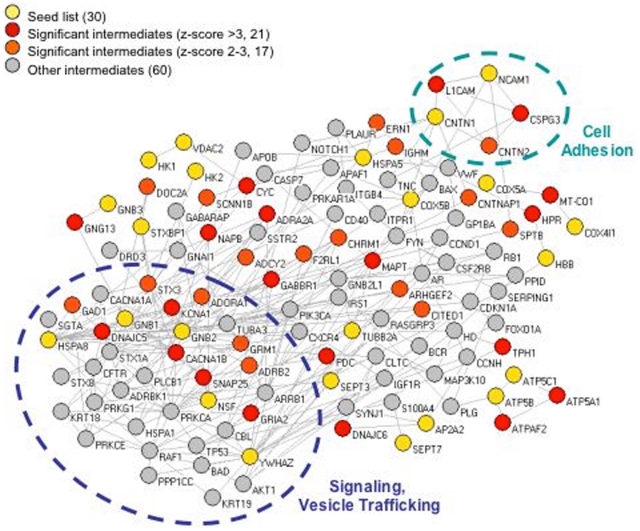
Network representation of proteins altered by morphine treatment. Proteins from the seed list (yellow) were connected via intermediates from the background dataset. Significant intermediates are shown in red (score>3) or orange (score between 2–3). The two clusters that were used to make predictions of morphine-regulated proteins are outlined.

To enrich the list and identify a network of proteins downregulated by morphine, we used the Genes2Networks [21] analysis (see Methods). Pairs of proteins from the seed list were connected via shared intermediates from a background dataset that was generated by combining databases of mammalian protein-protein interactions. This analysis resulted in a network containing 263 interactions among 28 proteins from the seed list, using 98 intermediates from the background dataset (Fig. 3). The clustering coefficient of this network is significantly higher (0.14) than the average clustering coefficient (0.086) computed from 100 shuffled networks created from the original topology (p<0.01). Using a binomial proportions test [21], [22], we found 38 intermediates from the background dataset to significantly (score>2) interact with proteins from the seed list. Of these, 21 proteins were considered highly significant (score>3).

Since functionally related nodes are likely to interact with each other while being more separate from the rest of the network [23], we sought to identify clusters within our network, consisting of areas in the network where nodes are more densely connected to each other. This allowed us to generate predictions of other proteins likely to be modulated by morphine. We identified 3 clusters with *k* = 4 and 10 clusters with *k* = 3 (Figure S1). These clusters are likely to consist of protein complexes or signaling pathways with specific presynaptic functions.

### Validation by Western blotting analysis

**Figure 4:**
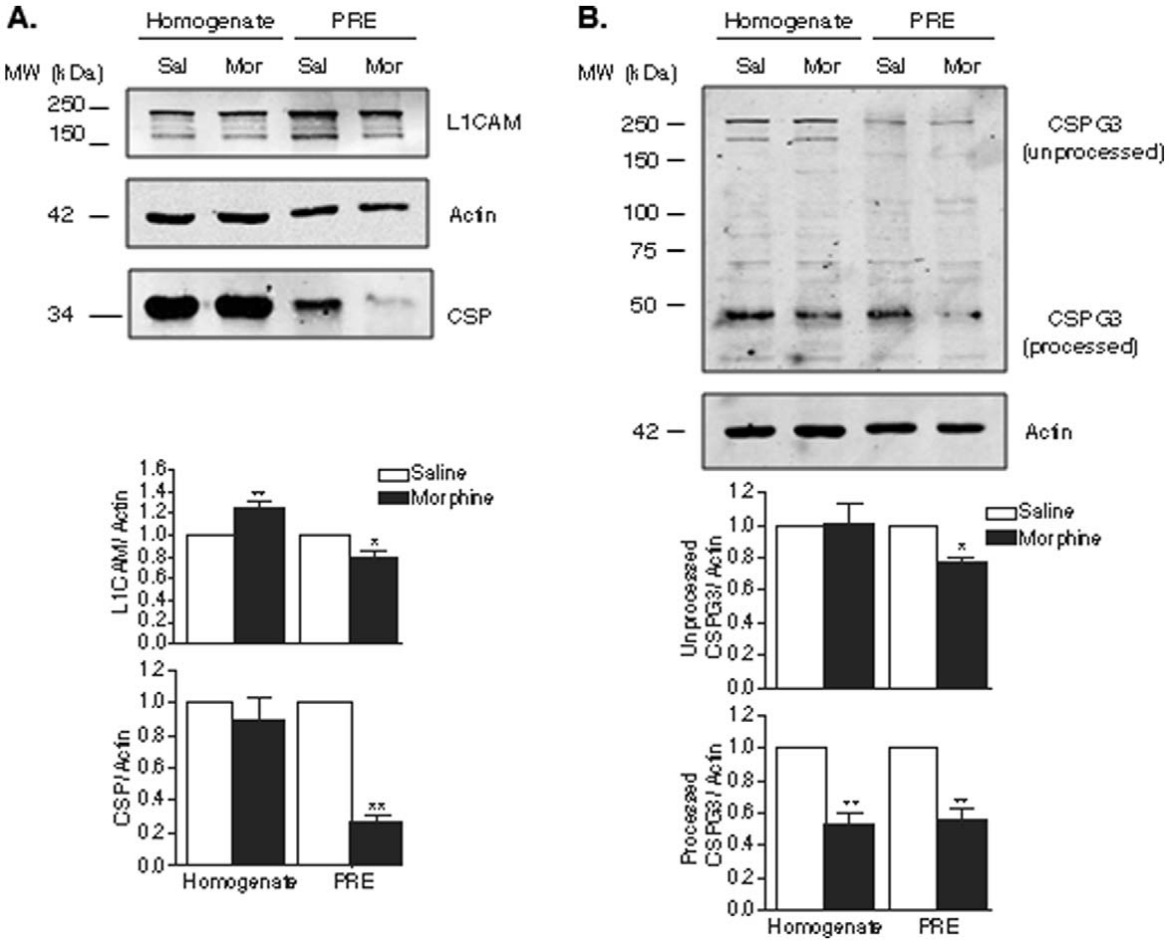
Validation of network analysis predictions. Using graph theory-based methods, L1CAM, neurocan (CSPG3), and CSP were predicted to be decreased at the presynapse by morphine treatment. Western blot analysis showed a decrease in (**A**) L1CAM, CSP, and (**B**) a lower molecular weight form of neurocan in the PRE fraction after chronic morphine administration. Protein levels were normalized to actin. A representative figure (out of 6 blots) is shown; the graph with statistical data is from multiple determinations **p<0.01 compared to saline treated (n = 6).

Some of the predictions generated by the cluster analysis were verified using Western blot analysis (Fig. 4). For this, we focused on two main clusters (outlined in Fig. 3): a small cluster consisting of 5 cell adhesion molecules (contactin 1 and 2, L1CAM, and neurocan or CSPG3); and the largest cluster, consisting of 36 proteins, including a large number of signaling and vesicle trafficking proteins, such as N-type calcium channels (CACNA1B) and cysteine string protein (CSP).

In the smaller cluster, morphine administration led to a decrease in the levels of L1CAM in the PRE fraction, but not in the total homogenate (Fig. 4A). A decrease in the processed form of neurocan or CSPG3 was also observed in the PRE fraction as well as in the homogenate fraction after morphine administration (Fig. 4B).

**Figure 5:**
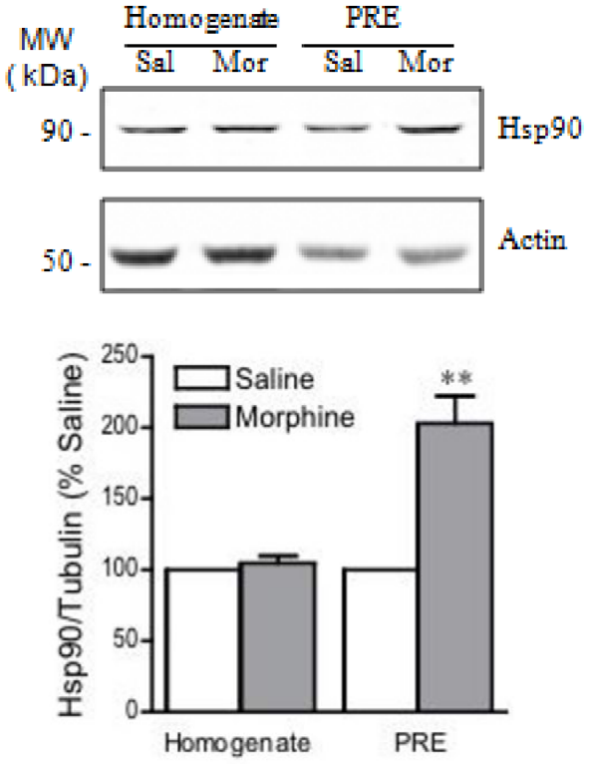
Hsp90 levels after chronic morphine administration. Western blot analysis showed an increase in the levels of Hsp90 in the PRE fraction after chronic morphine administration. No changes were observed in total homogenates suggesting redistribution of the protein rather than increases in gene expression changes. Protein levels were normalized to actin. **p<0.01 compared to saline treated (n = 6). A representative blot of 6 is shown.

In the largest cluster, we identified CSP as a significant intermediate (with the highest score of 6.45). Western blot analysis showed a significant decrease in the levels of this protein in the PRE fraction, but not in the total homogenate, after chronic morphine administration (Fig. 4A). To determine whether chronic morphine would also alter the levels of proteins that interact with CSP, we used Western blotting to measure Hsp90 levels. In contrast to CSP, chronic morphine led to an increase in Hsp90 levels in striatal PRE fractions but not in the total homogenate (Fig. 5).

### Naloxone-precipitated morphine withdrawal

**Figure 6:**
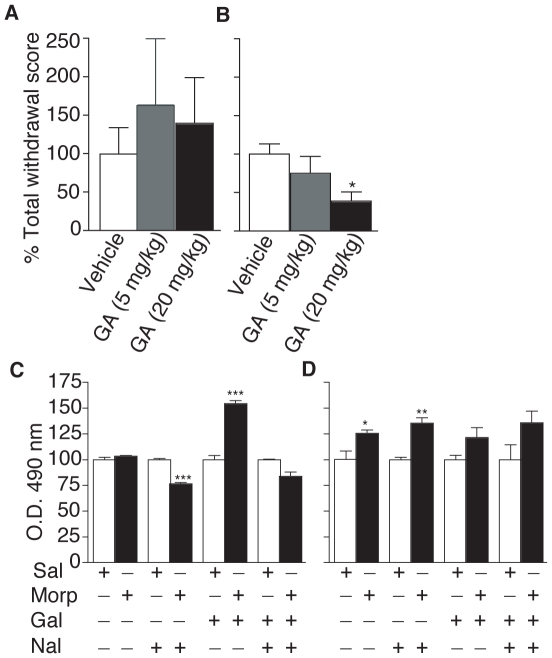
The effect of geldanamycin on naloxone-precipitated morphine withdrawal. (**A** & **B**) Mice treated chronically with saline (**A**) or morphine (**B**) were injected with either geldanamycin (GA; 5 or 20 mg/kg i.p.) or vehicle (20% DMSO in 0.9% saline) 2 h after the last injection, followed by naloxone (1 mg/kg s.c.) and withdrawal was evaluated for a period of 30 min as described in Methods. Geldanamycin (20 mg/kg) attenuates signs of morphine withdrawal. (**C** & **D**) Following behavioral analysis animals were sacrificed and Hsp90 levels were detected by ELISA in brain homogenates (**C**) and PRE fractions (**D**) as described in Methods. Data represents Mean ± SEM n = 4–12/group.

To assess the behavioral implications of the observed increase in Hsp90 levels, we examined the effect of Hsp90 inhibition on naloxone-precipitated morphine withdrawal (Fig. 6). In morphine-treated animals, administration of the selective Hsp90 inhibitor, geldanamycin, led to a dose-dependent decrease in somatic signs of withdrawal (Fig. 6B, Table 2). No significant effects were observed in animals chronically exposed to saline in the absence or presence of geldanamycin (Fig. 6A). After behavioral testing we used an ELISA assay to determine the levels of Hsp90 in brain homogenates and PRE fractions under different treatment conditions. As expected we observed an increase in Hsp90 levels in the PRE fraction but not in the total brain homogenate of animals treated chronically with morphine (Fig. 6C & D). This increase in Hsp90 levels in the PRE fraction was also observed following naloxone administration, although a decrease was seen in brain homogenates (Fig. 6C & D). Interestingly, the Hsp90 inhibitor, geldanamycin, significantly increased the levels of Hsp90 in brain homogenates but not in the PRE fraction of animals treated chronically with morphine (Fig. 6C & D). Taken together these results suggest that Hsp90 may play a role in dependence-associated behaviors.

**Table 2:** The effect of geldanamycin on naloxone-precipitated morphine withdrawal.

Withdrawal Signs	Vehicle(n = 12)	GA (5 mg/kg)(n = 4)	GA (20 mg/kg)(n = 7)
Rearing	55.33±7.87	32.86±13.45	22.33±9.38*
Jumping	37.18±6.49	19.5±14.66	2.86±1.72**
Forepaw Tremors	54.42±9.43	58.00±19.84	21.23±7.56
Teeth chattering	5.17±0.45	2.75±0.63*	1.57±0.48***
Ptosis	4.42±0.31	2.25±0.75*	3.14±0.51
Diarrhea	2.58±0.52	0.25±0.25*	0.42±0.30**

*p<0.05,

**p<0.01,

***p<0.001 Vehicle vs GA.

## Discussion

In this study, we undertook a quantitative subcellular proteomic analysis to study the effects of morphine on striatal presynaptic protein levels. We used a five-day paradigm of chronic intermittent escalating morphine administration that results in the development of significant opiate dependence [24], which is considered to be due to neuroadaptations in the mesolimbic system of the brain. This led to the identification of many proteins that may play a role in the synaptic plasticity events underlying the long-lasting and persistent effects of morphine in the brain.

### Vesicle trafficking

Our analysis showed morphine-induced downregulation of most of the proteins identified. Some of these are involved in various steps of synaptic vesicle trafficking, including vesicle priming (NSF), docking (syntaxin-binding protein 1 or Unc-18 homolog), and endocytosis (AP-2 alpha2), supporting previous reports of downregulation of SNARE complex formation [19], [20] and NSF upon morphine treatment [10]. Moreover, these results provide additional information on the proteins involved in the synaptic plasticity events thought to underlie addiction [25].

### Cytoskeleton-associated and cell adhesion

Our proteomics data showed morphine-induced decreases in several cytoskeleton-associated and cell adhesion molecules, including tubulin beta chain 15, septin 3 and 7, contactin 1, NCAM1, and opioid-binding cell adhesion molecule (OPCML or OBCAM) (Table 1). Western blot analysis of contactin 1 and NCAM 1 confirmed these results and showed that the decrease was specific to the PRE fraction and not to total homogenate, suggesting a redistribution of these proteins away from the presynaptic nerve terminal, rather than a global decrease in their expression levels. This is consistent with previous reports showing that continuous morphine treatment decreases NCAM1 levels in a synaptic membrane fraction^10^. Taken together, these results suggest a morphine-induced regulation of synapse architecture, which could play an important role in long-term synaptic plasticity. It is likely that other proteins at the presynapse are similarly translocated upon morphine treatment, although the mechanisms underlying this phenomenon remain unknown.

Similar results were observed for proteins predicted by our network analysis to be modulated by morphine. For instance, a decrease in processed neurocan was mostly seen in the PRE fraction. This, taken with the finding that decreases in L1CAM levels were seen only in PRE fraction and not in homogenate, further supports the novel concept of regulation of protein levels by redistribution of proteins from PRE to extra-synaptic areas (as opposed to changes in gene expression).

### Signaling

Previously, it was shown that long-term exposure to morphine leads to a decrease in the levels of several G protein subunits (α_i2_, α_i3_, β_1_, β_2_) in human neuroblastoma SH-SY5Y cells stably expressing the μ opioid receptor [26]. Our results show that chronic morphine treatment results in the downregulation of β_1_, β_2_, β_3_, and α_olf_ subunits of heterotrimeric G proteins, further supporting the modulation of signaling proteins by morphine. Interestingly, others have shown that this morphine-induced downregulation of Gβ seems to correlate with sensitization of adenylyl cyclase, a hallmark of opiate dependence [26].

### Chaperone

Another group of proteins, shown by our proteomics analysis to be downregulated by chronic morphine administration, includes molecular chaperones such as GRP 78, (Hsc70), and TCP1. Hsc70, a constitutively expressed protein, is a member of the 70 kDA heat shock protein family (Hsp70). It is enriched in the mammalian nervous system, particularly at synapses, where it plays a role in the folding of denatured proteins [27], [28]. It may also have a neuroprotective role that preserves synaptic function [28]. This protein also interacts with CSP, a member of the DnaJ (or Hsp40) protein family found on synaptic vesicles and clathrin coated vesicles in neurons [29]. Both proteins assemble into an enzymatically active chaperone complex that associates with Gβ, increasing its inhibition of N-type calcium channels [30]–[32], and this may be necessary for presynaptic neurotransmitter release [33]. This complex may also facilitate protein interactions at different stages of the vesicle trafficking cycle, regulating processes at all stages of exocytosis, including neurotransmitter synthesis and vesicle filling, docking, calcium entry, and vesicle fusion [34]. Our results showed a decrease in both Hsc70 (data not shown) and CSP in the PRE fraction upon morphine treatment, suggesting that these two proteins may have a critical effect on presynaptic function.

Having found significant morphine-induced changes in the levels of CSP and Hsc70, we sought to determine whether chronic morphine administration would also affect the levels of Hsp90, which is known to interact with these proteins under normal conditions [34]. Our results showed an increase in presynaptic Hsp90 levels after chronic morphine administration, suggesting a morphine-induced dissociation of Hsp90 from the interacting complex (i.e. from CSP and Hsc70) and recruitment to the presynaptic terminal that may result in its association with other yet-unidentified proteins at the presynapse. Additionally these studies suggest that although some proteins may not be detected by quantitative subcellular proteomic analysis, their presence can be deduced through the identification of interacting proteins by a combination of network and cluster analysis.

### Naloxone-precipitated morphine withdrawal

To further assess the functional implications of the observed increase in Hsp90 levels, we used a morphine withdrawal paradigm to determine whether this increase plays a role in morphine dependence. Our results showed that inhibition of Hsp90 by geldanamycin dose-dependently decreases somatic signs of morphine withdrawal, suggesting that Hsp90 may play an important role in dependence-associated behaviors and that its inhibition may alleviate symptoms of withdrawal in opiate-dependent subjects. Moreover, Hsp90 inhibitors may represent potential therapeutics to prevent the cellular adaptations to chronic morphine administration, since it was recently shown that inhibition of Hsp90 partially inhibits the adenylyl cyclase superactivation observed after chronic morphine administration [35]. Taken together, these studies suggest a provocative role for molecular chaperones in mediating presynaptic events that may underlie some of the long-lasting effects of morphine. In fact, there is evidence to suggest that Hsp90 may play an important role in neurotransmission [33], [36], suggesting that the changes observed after chronic morphine administration may be related to the role of Hsp90 in neurotransmission and not necessarily a general response to stress.

Morphine and other addictive drugs produce significant and persistent adaptations at the synaptic level that may underlie their long-lasting addictive potential [25]. We previously showed the powerful potential that an integrated proteomics and computational approach has to make biologically relevant predictions that can be tested experimentally [22]. Here we used this approach to gain a better understanding of the presynaptic proteins, signaling pathways, and complexes that are regulated by morphine and to identify potential targets for treatment of opiate dependence and addiction (Figure S2). One of the major substrates of the molecular and cellular mechanisms of opiate addiction is the striatum, which has been implicated in reward, habit learning, craving and relapse. This information on chronic morphine-induced changes in striatal presynaptic proteins constitutes an important first step to guide future studies on the roles of these proteins in addiction. Furthermore, this study demonstrates that such an approach using proteomic techniques in combination with computational graph theory analysis, allows a unique and more complete understanding of neurobiological networks at the presynapse and their regulation.

## Materials and Methods

### Animals and Drug Treatment

Protocols involving animals were conducted in accordance with the recommendations set forth in the *Guide for the Care and Use of Laboratory Animals* by the National Institutes of Health, and were approved by the Institutional Animal Care and Use Committee at Mount Sinai School of Medicine (Protocol Number 02-0805). Adult male Sprague-Dawley rats (200–250 g) or C57BL/6 mice (25–30 g) were maintained on a 12-h light/dark cycle and provided with food and water *ad libitum*. Animals were allowed to acclimatize to their environment for a week prior to drug administration. Morphine sulfate (Sigma, St. Louis, MO, USA) was prepared in 0.9% sterile isotonic saline. Animals were injected intraperitoneally (i.p.) with saline or morphine for 5 days. In the latter case, morphine was injected in escalating doses (5, 10, 15, 20, 25, 30, 35, 40 and 50 mg/kg) every 12 h. This intermittent, escalating dose paradigm of morphine administration results in the development of morphine dependence and withdrawal, and is refered to as chronic morphine aministration in the field. Animals were sacrificed 2 h after the last injection.

### Subcellular Fractionation

After chronic morphine treatment, animals were sacrificed by decapitation and the brains rapidly removed. Isolation of a presynaptic (PRE) fraction was performed as previously described [22]. The striata from 3 saline- or morphine-treated rats were combined and homogenized in 3 ml of 0.32 M sucrose, 0.1 mM CaCl_2_, with 30 µl each of protease and phosphatase inhibitor cocktails (Sigma, St. Louis, MO) at 4°C. All of the following fractionation steps were carried out at 4°C unless otherwise specified. The homogenate was brought to a final concentration of 1.25 M sucrose by the addition of 2 M sucrose (12 ml) and 0.1 mM CaCl_2_ (5 ml). The homogenate was then placed in a 40 ml ultracentrifuge tube and overlaid with 10 ml 1 M sucrose, 0.1 mM CaCl_2_. The gradients were centrifuged at 100,000 g for 3 h. The synaptosomal fraction (4–5 ml) was collected at the 1.25 M/1 M interface. To obtain synaptic membranes, the synaptosomal fraction was brought to a volume of 35 ml with 20 mM Tris-Cl pH 6, 0.1 mM CaCl_2_, containing 1% Triton X-100 (TX-100) and 350 µl each of protease and phosphatase inhibitor cocktails, mixed for 20 min, and centrifuged at 40,000 g for 20 min. The pellet containing the isolated synaptic membranes was collected. To separate a presynaptic fraction from the post-synaptic density (PSD), the pellet was resuspended in 20 ml of 20 mM Tris-Cl pH 8, 1% TX-100, 0.1 mM CaCl_2_. The mixture was again mixed for 20 min, and centrifuged at 40,000 g for 20 min. The supernatant was removed and concentrated to 1 ml using an Amicon Ultra-15 filter (5,000 MW cut-off, Millipore, Bedford, MA). The concentrate was precipitated with 9 ml of acetone by incubation at −20°C for 12 h, and centrifugation at 15,000 g for 30 min. The resulting pellet, containing the PRE fraction, was stored at −80°C until use. We have previously shown that the PRE fraction, isolated in this manner, is enriched in presynaptic proteins and excludes proteins that are enriched in the PSD fraction [22].

### Tryptic Digestion and Isotopic Labeling

Labeling experiments were performed as described elsewhere [19], [20], using three independent pools of PRE fractions isolated from three rats each. The pellet containing the PRE fraction was resuspended and solubilized in 0.1% SDS to a final concentration of 1 µg/µL. 30 µg of protein extract were reduced using a 1,4-dithiothreitol (DTT) solution (200 mM DTT in 100 mM NH_4_HCO_3,_ pH 8–10) at 40°C for 1 h. The reduced proteins were alkylated using 1 M iodoacetamide (IAM) in 100 mM NH_4_HCO_3_ for 40 min at room temperature (RT) in the dark. Unreacted IAM was quenched with the DTT solution for 1 h. The alkylated proteins were precipitated with cold acetone overnight, and the precipitate was pelleted by centrifugation (15000 rpm, 40 min, 4°C), then dissolved in digestion buffer (50 mM NH_4_HCO_3_, 1 M urea, pH 8–10), with 0.1 µg of trypsin (Sigma) at 40°C for 3 h. The pH of the tryptic digests was then adjusted to 9–10 with 1 M NaOH. For quantification, covalent modification of the tryptic peptides was performed by adding 2 µL of light (2 M succinic anhydride in DMSO) or heavy (2 M succinic [^2^H_4_] anhydride in DMSO) label [37]. The samples were vortexed and centrifuged, then incubated for 15 min at RT. The pH of the solutions was readjusted to 9–10. The labeling procedure was repeated 4 times with subsequent adjustment of pH. Remaining unreacted isotopic labels were quenched using 10 µL of 2.5 M glycine for 1 h at RT. The light and heavy isotope-labeled samples were then combined and desalted using a PepClean™ C18 spin column (Pierce), as per the manufacturer's protocol, and peptides eluted using 70% aqueous acetonitrile solution. The solvent in the eluate was removed using a vacuum centrifuge, and the residue containing the peptides was reconstituted in 20 µL of solvent A (5% aqueous acetonitrile with 0.1% FA and 0.01% TFA in water). 10 µL aliquots from each sample were used for LC-MS/MS analysis as described below.

### Mass Spectrometry

Peptide separation and MS analysis were performed using a capLC™ (Micromass, UK) system coupled to an HCTUltra-PTM Discovery system ion-trap mass spectrometer (Bruker Daltonics, Billerica, MA, USA) equipped with an electrospray ionization source. The sample was injected using a manual injector (Valco Instruments Co, Inc., TX, USA) and loaded onto a trap column (PepMap™, C18, 5 µm, 100 Å, LC Packings) using solvent A and washed for 5 min. The trapped peptides were then eluted in the reverse direction onto a reverse phased capillary column (LC Packings™ 300 µm i.d.×15 cm, C18 PepMap100, 100 Å) using a solvent gradient at a flow rate of 2 µL/min. The solvent gradient was generated using solvent A and solvent B (95% aqueous acetonitrile with 0.1% FA and 0.01% TFA in water). The 70 min gradient run for LC separation included 3 steps: 5–80% solvent B in 15–55 min (linear); 80% solvent B for 55–60 min (isocratic); 80–5% solvent B in 60–65 min (linear). MS data acquisition and the subsequent MS/MS of selected peaks were performed in a data-dependent manner using the Esquire software (Bruker Daltonics). For each MS scan, three peptides were selected to be fragmented, for 300–500 ms, based on their charge (preferably +2) and intensity. Dynamic exclusion of previously fragmented precursor ions was set to 2 spectra for a period of 60 s. The MS and MS/MS scan were performed in the range of m/z 300–1500 and 50–2000 respectively.

### Protein Identification and Quantification

The data were processed using the Data analysis software (Bruker Daltonics). MS data obtained between 20 and 45 min of the LC run was searched for compounds using an automated search option. The short-listed compounds with their respective MS/MS scans were directly exported to Biotools software (Bruker) for database searching using an in-house Mascot database search engine (Matrix Science). The mass tolerance of this study was set at 0.1% for MS and 0.5 Da for MS/MS. The search parameters included fixed modifications for cysteine (carbamidomethyl) and variable modifications for methionine (Met-oxidized), succinic anhydride or succinic [^2^H_4_] anhydride modified lysine and N-terminal amines. With Mascot, every tandem mass spectrum was assigned a list of matching database peptide sequences accompanied by a score representing the quality of each sequence identification. A Mascot score of 50 is commonly used as a cut-off for 95% confident identification. Only proteins identified with a Mascot score ≥60 for every peptide were considered for further analysis (for further experimental details on these measurements see references [37], [38]). For quantification, peak pairs with mass differences of 2, 4 or 8 Da, a retention time window of 30 s, and a signal-to-noise intensity >10 were listed using Data Analysis software, along with their peak intensity ratios and retention times. The morphine/saline ratio for each protein reflects the average of ratios obtained for each of the isotopically labeled tryptic peptides identified. During data acquisition only one of the labeled peptides was selected for fragmentation. In several cases, peptide peak pairs were manually identified and quantified.

### Integration of Proteomics Data into a PPI Network

The proteins altered by morphine treatment were placed in the context of signaling pathways and protein complexes using the software tool Genes2Networks (http://actin.pharm.mssm.edu/genes2networks) [21]. Genes2Networks integrates the contents of 10 mammalian binary interaction network datasets: BIND [39], DIP [40], [41], HPRD [42], IntAct [43], Ma'ayan [44], MINT [45], Stelzl [46], Vidal [47], PDZBase [48], and PPID [49]. To this, we added our own presynaptic PPI network dataset, http://amp.pharm.mssm.edu/presynaptome [22]. The consolidated background dataset before filtering contained 11,053 nodes and 44,985 links, with nodes representing the proteins, and links representing direct protein-protein interactions. To decrease the level of false positives resulting from high-throughput experiments, the dataset was filtered to exclude interactions originating from articles that provided five or more interactions. The final, filtered background dataset contained 2,788 nodes and 19,695 links. The proteins we identified as altered in the presynapse were connected using a maximum of two intermediates from the background dataset (path length of two nodes and three links). The resultant network was visualized using the signaling network analysis and visualization integrator (SNAVI) software package [50].

### Network Analysis – Significant Intermediates and Clustering

A binomial proportions test was used to evaluate the significance of interactions between proteins from the background dataset with the seed list. The z-sore (referred to as “score”) for each protein from the background dataset was computed as described previously [21], [22]. A higher score for a protein would indicate that the number of its interactions with proteins from our experimentally determined seed list is significantly enriched compared with the number of its interactions with other proteins from the background network. In this analysis, we considered proteins with a score between 2–3 to be significant interactors with proteins from the seed list, and proteins with a score between >3 to be highly significant. Cfinder (http://www.cfinder.org/) [51] was used to locate and visualize clusters in the PPI network containing the proteins altered by morphine treatment and the intermediates connecting them. CFinder uses the clique percolation method to locate *k*-clique percolation clusters in the network [52]. A *k*-clique is defined as a complete, or fully connected, subgraph on *k* nodes within the cluster. Two *k*-cliques are considered adjacent if they share *k*-1 nodes, i.e. if they differ only in a single node. A *k*-clique percolation cluster consists of a maximal *k*-clique-connected subgraph, i.e. the union of all *k*-cliques that can be reached via chains of adjacent *k*-cliques, and the links in these cliques. This method allows the identification of overlapping clusters, such that a single node can belong to more than one cluster.

### Western Blotting

10 µg of protein from each fraction were resolved in 7.5% SDS-PAGE and analyzed by Western blots with the following antibodies: CSP (1∶3000, Stressgen, Victoria, BC), α-contactin (1∶1000, gift from J Salzer, NYU), neurocan (1∶2000, gift from R. Margolis, NYU) L1CAM (1∶5,000, gift from D. Felsenfeld, MSSM), NCAM1 (gift from G. Phillips, MSSM), Hsp90 (1∶10,000, Stressgen), Hsp70 (1∶10,000, Stressgen), actin (1∶10,000, Sigma).

### Naloxone-Precipitated Morphine Withdrawal

C57BL/6 adult male mice (20–25 g) were injected with chronic morphine or saline (i.p.) as described above (n = 4–12 animals per group). Two hours after the last injection, animals were injected with geldanamycin (5 or 20 mg/kg i.p.) or vehicle (20% DMSO in 0.9% saline), followed by naloxone (1 mg/kg s.c.). After naloxone injection, six somatic signs of withdrawal were evaluated for a period of 30 min. Three signs (jumping, rearing and forepaw tremors) were counted and three signs (teeth chattering, ptosis and diarrhea) received a score of 1 for every 5-min interval in which it was present. After behavioral tests were complete, animals were sacrificed, brains were extracted and used to prepare homogenate and PRE fractions as described above. Hsp90 levels in these fractions were determined by ELISA as described previously [53] using 10 µg protein, 1∶2000 dilution of Hsp90 antibody and 1∶2000 dilution of HRP conjugated anti-rabbit antibody.

## Supporting Information

Figure S1 Clusters identified in the network of proteins altered by morphine treatment. Clusters were identified and visualized using CFinder, which uses the clique percolation method to identify overlapping clusters. A total of 13 overlapping clusters were identified in the network: 3 clusters with k = 4 and 10 clusters with k = 3.
(TIF)

Figure S2 A flow chart summarizing the process of proteomic data analysis and computational predictions. Simplified schematic of the approaches used to identify morphine-regulated presynaptic proteins by quantitative proteomics, and to map potential presynaptic signaling pathways and protein complexes by graph theory. These were then used to predict novel morphine-regulated proteins. F = forward labeling, R = reverse labeling. The number of proteins quantified is indicated in parentheses.
(TIF)

Table S1 Each sample represents a pool of 3 striatal PRE fractions from saline- and morphine-treated rats. In total, 175 unique proteins were identified, and 143 of these were quantified. Proteins identified were those with Mascot scores ≥60.
(DOC)

Table S2 Analysis of MS/MS spectra led to the identification of 175 proteins, 143 of which were quantified by determining the peak intensity of the labeled peptides. Only 30 of these proteins were robustly and consistently altered by morphine treatment.
(DOC)
