# Supplementary figures and images for: Chronic Morphine Alters the Presynaptic Protein Profile: Identification of Novel Molecular Targets Using Proteomics and Network Analysis
Source: PLoS One. 2011 Oct 17;6(10):e25535. doi: 10.1371/journal.pone.0025535 (PMC3197197; doi:10.1371/journal.pone.0025535)

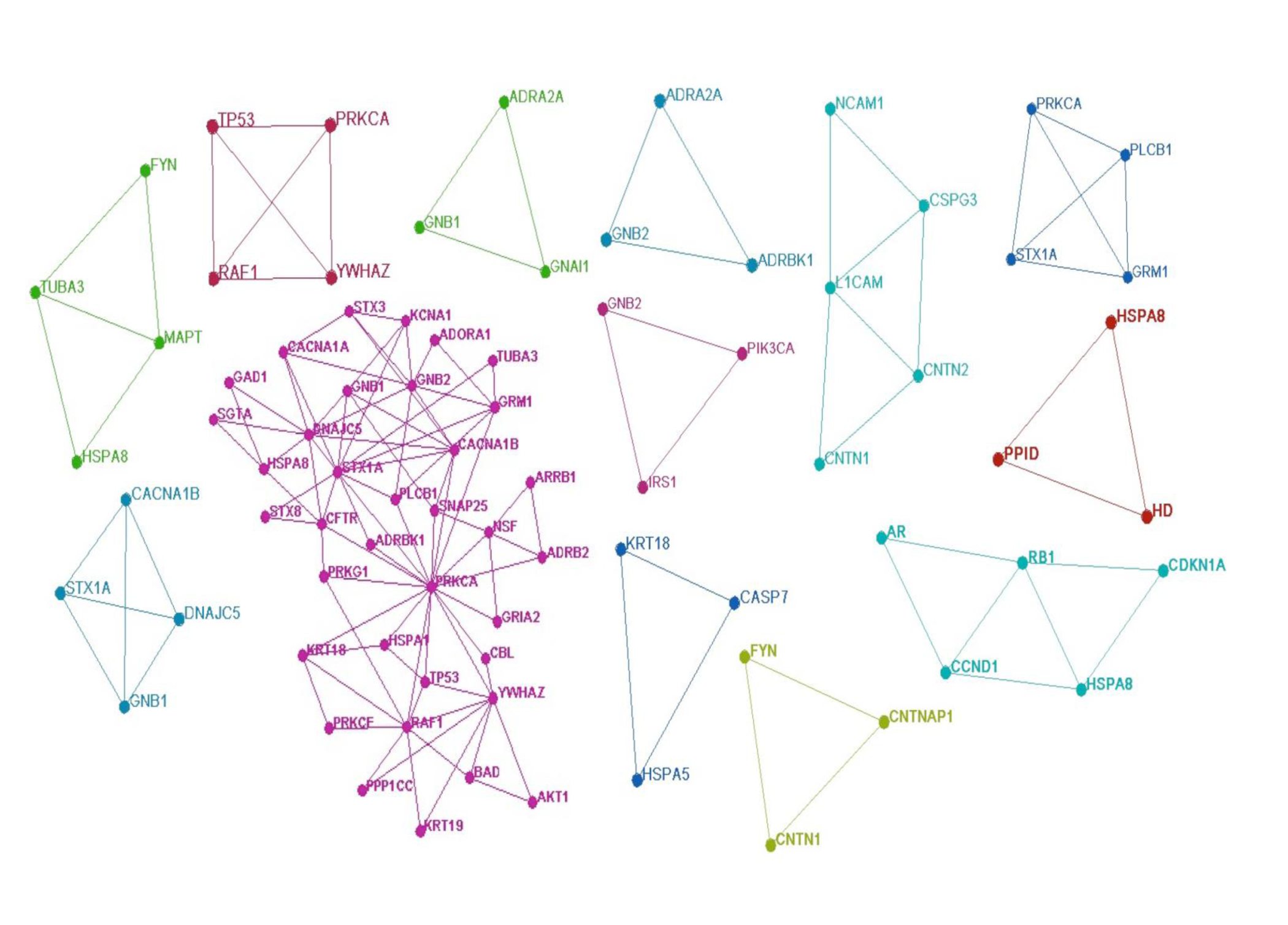

Supplement: Figure S1 — Clusters identified in the network of proteins altered by morphine treatment. Clusters were identified and visualized using CFinder, which uses the clique percolation method to identify overlapping clusters. A total of 13 overlapping clusters were identified in the network: 3 clusters with k = 4 and 10 clusters with k = 3. (TIF) [file pone.0025535.s001.tif]

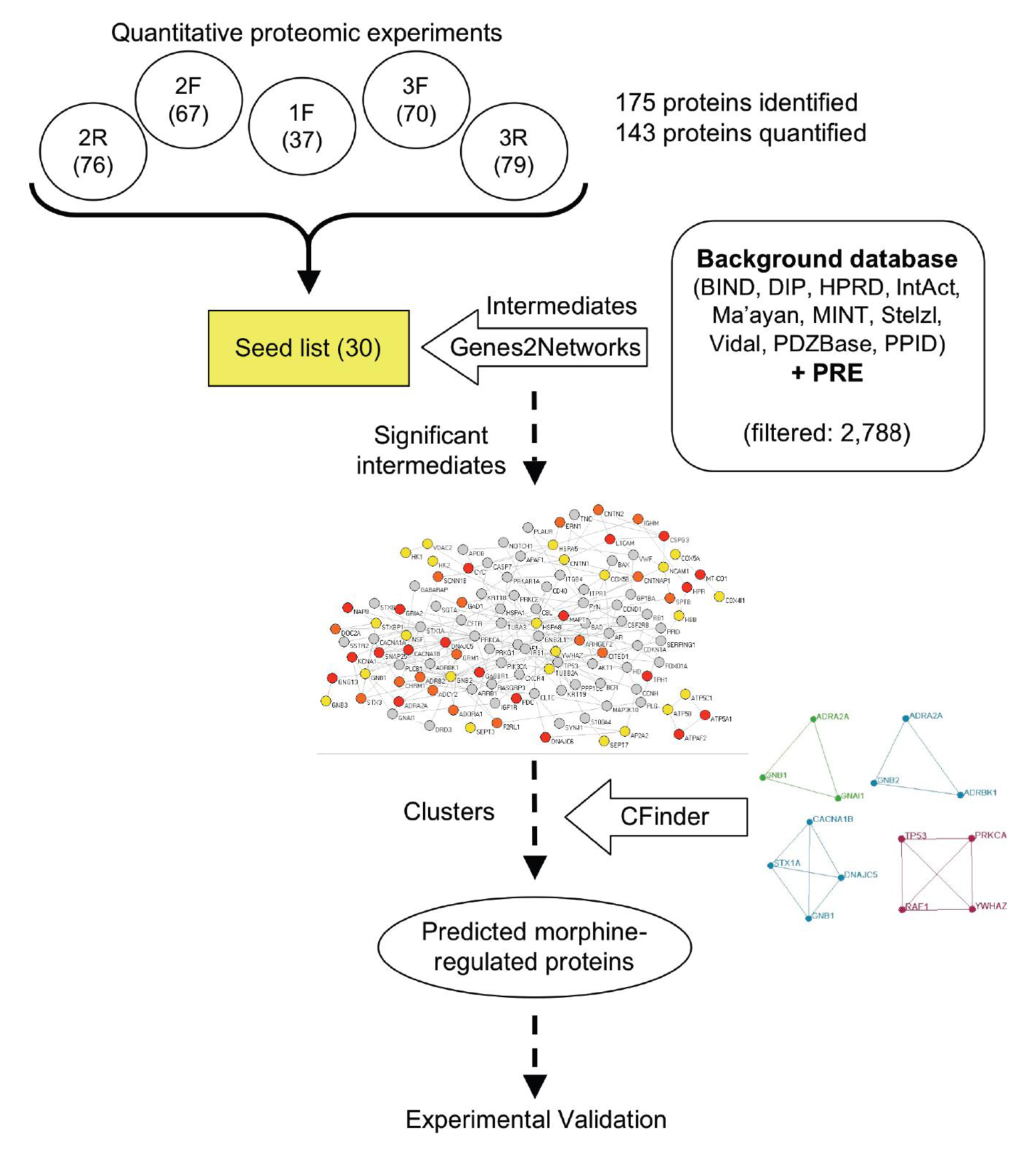

Supplement: Figure S2 — A flow chart summarizing the process of proteomic data analysis and computational predictions. Simplified schematic of the approaches used to identify morphine-regulated presynaptic proteins by quantitative proteomics, and to map potential presynaptic signaling pathways and protein complexes by graph theory. These were then used to predict novel morphine-regulated proteins. F = forward labeling, R = reverse labeling. The number of proteins quantified is indicated in parentheses. (TIF) [file pone.0025535.s002.tif]
