# Supplementary material for: Chronic Morphine Alters the Presynaptic Protein Profile: Identification of Novel Molecular Targets Using Proteomics and Network Analysis
Source: PLoS One. 2011 Oct 17;6(10):e25535. doi: 10.1371/journal.pone.0025535 (PMC3197197; doi:10.1371/journal.pone.0025535)
Supplement: Table S1 — Each sample represents a pool of 3 striatal PRE fractions from saline- and morphine-treated rats. In total, 175 unique proteins were identified, and 143 of these were quantified. Proteins identified were those with Mascot scores ≥60. (DOC) [file pone.0025535.s003.doc]

**Table S**1. Summary of proteomic experiments using differential isotopic labeling and LC-MS/MS.

| **Sample** | **Labeling** | **Proteins Identified** | **Proteins Quantified** | **Mean Mor/Sal Ratio** | **Std. Dev.** | **Std. Error** |
| --- | --- | --- | --- | --- | --- | --- |
| 1 | Forward | 48 | 37 | 0.92 | 0.27 | 0.043 |
| 2 | Forward | 80 | 67 | 0.97 | 0.261 | 0.032 |
| 2 | Reverse | 84 | 76 | 0.75 | 0.349 | 0.04 |
| 3 | Forward | 77 | 70 | 0.91 | 0.234 | 0.028 |
| 3 | Reverse | 86 | 79 | 0.75 | 0.29 | 0.033 |
