# Supplementary material for: Chronic Morphine Alters the Presynaptic Protein Profile: Identification of Novel Molecular Targets Using Proteomics and Network Analysis
Source: PLoS One. 2011 Oct 17;6(10):e25535. doi: 10.1371/journal.pone.0025535 (PMC3197197; doi:10.1371/journal.pone.0025535)
Supplement: Table S2 — Analysis of MS/MS spectra led to the identification of 175 proteins, 143 of which were quantified by determining the peak intensity of the labeled peptides. Only 30 of these proteins were robustly and consistently altered by morphine treatment. (DOC) [file pone.0025535.s004.doc]

**Table S2. Proteins identified and quantified in saline- and morphine-treated striatal PRE fractions by differential isotopic labeling and MS/MS.**

| **Protein Name** | **UniProt Acc. #** | **Score** | **# Pept. Ident.** | **# Pept. Labeled** | **Mean M/S Ratio** | **Sample** |
| --- | --- | --- | --- | --- | --- | --- |
| Actin, cytoplasmic 1 | P60711 | 448 | 14 | 6 | 0.7 | 2F, 2R, 3R |
| Actin, cytoplasmic | P63259 | 378 | 20 | 8 | 0.85 | 1 |
| Actin, gamma-enteric smooth muscle | P63269 | 321 | 14 | 5 | 0.8 | 2F, 2R, 3F, 3R |
| Actn1 protein | Q6GMN8 | 77 | 4 | 1 | 1.33 | 1 |
| Alpha actinin 4 | Q6P786 | 73 | 5 | 1 | 1.33 | 1 |
| Adenylyl cyclase type VI | Q03343 | 72 | 3 |  |  | 2F |
| Fructose-bisphosphate aldolase A | P05065 | 607 | 19 | 10 | 0.78 | 1, 2F, 2R, 3F, 3R |
| Fructose-bisphosphate aldolase B | P00884 | 81 | 5 | 2 | 0.98 | 2F |
| Fructose-bisphosphate aldolase C | P09117 | 90 | 4 | 1 | 0.78 | 2F, 2R |
| Ankyrin | P97582 | 88 | 4 | 1 | 1.67 | 3F |
| Annexin A1 | P07150 | 74 | 4 | 2 | 0.67 | 2R |
| AP-2 alpha-2 | P18484 | 166 | 8 | 2 | 0.83 | 1, 2F, 3F, 3R |
| AP-2 beta-1 | P62944 | 108 | 6 | 3 | 0.88 | 1, 2F, 2R, 3F |
| AP-2 sigma-1 | P62744 | 59 | 1 | 1 | 1.1 | 1 |
| Aa1064 | Q7TMA5 | 53 | 16 |  |  | 1 |
| Na/K-transporting ATPase alpha-1 chain | P06685 | 69 |  |  |  | 3R |
| Na/K-transporting ATPase alpha-3 chain | P06687 | 60 | 5 |  |  | 2F |
| ATP synthase alpha chain, mitochondrial | P15999 | 928 | 30 | 13 | 0.85 | 1, 2F, 2R, 3F, 3R |
| ATP synthase subunit beta, mitochondrial | P10719 | 880 | 29 | 11 | 0.94 | 1, 2F, 2R, 3F, 3R |
| ATP synthase gamma chain, mitochondrial | P35435 | 158 | 6 | 3 | 0.95 | 2R, 3F, 3R |
| ATP synthase B chain, mitochondrial | P19511 | 117 | 4 | 2 | 0.84 | 2F, 2R, 3F, 3R |
| ATP synthase D chain, mitochondrial | P31399 | 83 | 2 | 1 | 1.2 | 3R |
| ATP synthase O subunit, mitochondrial | Q06647 | 179 | 6 | 3 | 0.92 | 2F, 2R, 3F, 3R |
| Vacuolar ATPase 116 kDa subunit a isoform 1 | P25286 | 147 | 6 | 3 | 0.9 | 2R, 3F, 3R |
| ATPase, H+ transporting, V0 subunit D isoform 1 | Q5M7T6 | 150 | 3 | 2 | 0.93 | 2F, 2R, 3F, 3R |
| Vacuolar ATPase subunit B, brain isoform | P62815 | 106 | 3 | 1 | 0.73 | 2R |
| ATPase, H+ transporting, V1 subunit E isoform 1 | Q6PCU2 | 50 | 2 |  |  | 2F |
| T-complex protein 1 subunit alpha | P28480 | 90 | 5 | 2 | 0.78 | 2F, 2R, 3R |
| Serine/threonine-protein kinase MRCK alpha | O54874 | 75 | 6 | 1 | 0.94 | 2R, 3F, 3R |
| Creatine kinase, ubiquitous mitochondrial | P25809 | 121 | 5 | 2 | 0.94 | 2F, 2R, 3F, 3R |
| CLIP-associating protein CLASP2 | Q99JD4 | 124 | 9 | 2 | 0.85 | 1, 2F, 2R, 3F |
| Clathrin heavy chain | P11442 | 540.4 | 21 | 9 | 0.93 | 1, 2F, 2R, 3F, 3R |
| Contactin-1 | Q63198 | 87.34 | 5 | 2 | 0.73 | 2R, 3F, 3R |
| COP9 signalosome complex subunit 2 | P61203 | 56 | 5 |  |  | 1 |
| Cytochrome c oxidase subunit 4 isoform 1 | P10888 | 110 | 5 | 2 | 0.91 | 1, 2F, 2R, 3F, 3R |
| Cytochrome c oxidase subunit Va | P11240 | 280.3 | 8 | 5 | 1.05 | 2F, 2R, 3F, 3R |
| Cytochrome c oxidase subunit Vb | P12075 | 80 | 3 | 2 | 1.04 | 2F, 2R, 3F, 3R |
| Dihydropyrimidinase-related protein 1 | Q62950 | 82 | 5 | 2 | 0.94 | 2F |
| Citrate synthase, mitochondrial | Q8VHF5 | 118.22 | 5 | 3 | 0.81 | 2F, 2R, 3F, 3R |
| Alpha-S2-casein-like A | P02667 | 52 | 5 |  |  | 2F |
| Basement membrane-associated chondroitin proteoglycan Bamacan | P97690 | 52 | 7 |  |  | 2F |
| Cytochrome c, somatic | P62898 | 72.14 | 2 | 1 | 0.57 | 2F, 3F |
| Disabled homolog 2-interacting protein | Q6P730 | 80 | 10 | 1 | 0.97 | 2F, 2R |
| Dihydrolipoyllysine-residue acetyltransferase component of pyruvate dehydrogenase complex | P08461 | 138 | 5 | 3 | 0.99 | 2F, 2R, 3F, 3R |
| Dihydrolipoyl dehydrogenase | Q6P6R2 | 94.32 | 4 | 2 | 0.51 | 2R, 3R |
| Dihydrolipoamide S-succinyltransferase | Q01205 | 135.54 | 6 | 2 | 0.78 | 1, 2F, 3F, 3R |
| Dynamin-1 | P21575 | 272 | 14 | 8 | 0.91 | 1, 2F, 2R, 3F, 3R |
| Dynamin-3 | Q08877 | 101.62 | 9 | 4 | 0.69 | 1, 2R |
| Dihydropyrimidinase-related protein 2 | P47942 | 67 | 5 | 1 | 0.55 | 1, 2F, 3R |
| Elongation factor 1-alpha 1 (EF-1-alpha-1) | P62630 | 64 | 3 | 1 | 1.3 | 1 |
| Elongation factor 1-alpha 2 (EF-1-alpha-2) | P62632 | 58 | 3 |  |  | 1, 2F |
| Elongation factor 1-gamma (EF-1-gamma) | Q68FR6 | 51 | 3 |  |  | 2F |
| Fumarate hydratase, mitochondrial precursor | P14408 | 134 | 5 | 2 | 0.85 | 2F, 3F, 3R |
| Fumarate hydratase 1 | Q5M964 | 105.93 | 3 | 2 | 0.94 | 2R |
| Filamin-A-interacting protein 1 (FILIP) | Q8K4T4 | 71 | 9 | 1 | 0.84 | 3R |
| GTP-binding protein Golf alpha subunit | Q80WZ0 | 159 | 7 | 2 | 0.83 | 1, 2F, 2R, 3F, 3R |
| Glyceraldehyde-3-phosphate dehydrogenase | P04797 | 61 | 6 |  |  | 1 |
| Glutamate dehydrogenase 1, mitochondrial | P10860 | 97 | 4 | 2 | 1.26 | 2R, 3R |
| Glutamine synthetase | P09606 | 125 | 6 | 3 | 0.74 | 2F, 3F, 3R |
| G protein alpha-12 subunit | Q63210 | 150 | 9 | 2 | 0.88 | 1, 2F, 2R, 3F, 3R |
| G protein alpha 13 | Q6Q7Y5 | 105.69 | 7 | 1 | 0.8 | 1, 2R |
| G protein G(k) subunit alpha (G(i) alpha-3) | P08753 | 100 | 4 |  |  | 1 |
| G protein G(o) subunit alpha 1 | P59215 | 292.63 | 14 | 5 | 0.8 | 1, 2F, 2R, 3F |
| G protein G(o) subunit alpha 2 | P30033 | 218 | 8 | 4 | 0.66 | 3R |
| G protein G(s) subunit alpha isoform short |  | 95 | 6 | 1 | 1.05 | 2F |
| Gprotein G(t), alpha-3 subunit | P29348 | 117.06 | 6 | 1 | 1.02 | 1, 3F |
| G protein G(I)/G(S)/G(T) subunit beta 1 | P54311 | 321.6 | 11 | 6 | 0.82 | 1, 2F, 2R, 3F, 3R |
| G protein G(I)/G(S)/G(T) subunit beta 2 | P54313 | 216 | 7 | 3 | 0.57 | 1, 3R |
| G protein G(I)/G(S)/G(T) subunit beta 3 | P52287 | 135 | 5 | 3 | 0.64 | 1, 2F, 2R, 3R |
| Glucose phosphate isomerase | Q6P6V0 | 99 | 5 | 1 | 0.73 | 2F, 2R, 3R |
| Glutamate receptor-interacting protein 2 | Q9WTW1 | 52 | 4 |  |  | 1 |
| Hemoglobin subunit alpha-1/2 | P01946 | 163 | 5 | 3 | 0.96 | 2F, 3F |
| Histone H1.2 | P15865 | 91.11 | 8 | 1 | 0.78 | 2R, 3F, 3R |
| Histone H1t | P06349 | 82 | 4 | 1 | 0.73 | 2R, 3F, 3R |
| Hexokinase 2 | P27881 | 156.08 | 5 | 1 | 0.59 | 1, 2F, 2R, 3R |
| Heat shock protein 86 | Q91XW0 | 130 | 9 | 2 | 0.99 | 2F, 2R, 3F, 3R |
| Heat shock 70 kDa protein 1A/1B | Q07439 | 86.86 | 6 | 1 | 0.87 | 1, 2F, 3F |
| Heat shock 70 kDa protein 1L | P55063 | 74.18 | 5 | 1 | 0.76 | 2F, 3F |
| dnaK-type molecular chaperone hst70 | P14659 | 107.13 | 5 | 2 | 0.81 | 3F, 3R |
| 78 kDa glucose-regulated protein precursor | P06761 | 111.68 | 8 | 1 | 0.74 | 2F, 2R, 3F, 3R |
| Heat shock cognate 71 kDa protein | P63018 | 227.34 | 10 | 4 | 0.8 | 1, 2F, 2R, 3F, 3R |
| Heat shock 90kDa protein 1, beta | Q66H55 | 113 | 8 | 2 | 0.85 | 2F, 2R, 3R |
| Isocitrate dehydrogenase [NAD] subunit alpha | Q99NA5 | 211.89 | 7 | 3 | 0.82 | 1, 2F, 2R, 3F, 3R |
| Kdr protein | Q5PQU0 | 55 | 4 |  |  | 1 |
| SLP-76 adaptor ptotein | Q920L0 | 59 | 5 |  |  | 2F |
| LOC298795 protein | Q68FS0 | 81 | 3 | 1 | 1.16 | 2F |
| Limbic system-associated membrane protein | Q62813 | 71.01 |  |  |  | 2R |
| Beta-glo | Q6PDU6 | 146 | 4 | 2 | 1.05 | 2F, 2R |
| Cytochrome c oxidase subunit 2 | P00406 | 133.76 | 6 | 3 | 0.95 | 2F, 2R, 3F |
| Neural cell adhesion molecule 1 | P13596 | 187.18 | 10 | 4 | 0.84 | 1, 2F, 2R, 3F, 3R |
| NADH dehydrogenase 1 alpha subcomplex 10-like | Q80WE0 | 73.29 | 2 | 1 | 1.11 | 3F |
| NADH dehydrogenase (Ubiquinone) Fe-S protein 1 | Q66HF1 | 83.02 | 2 | 1 | 1.15 | 2F, 2R, 3F |
| NADH dehydrogenase (Ubiquinone) flavoprotein 1 | Q5XIH3 | 55 | 4 |  |  | 2F |
| NADH dehydrogenase [ubiquinone] flavoprotein 2 | P19234 | 73 | 5 | 1 | 1.1 | 2F |
| N-ethylmaleimide sensitive factor | Q9QUL6 | 123 | 9 | 2 | 0.6 | 1, 3F, 3R |
| Opioid-binding protein/cell adhesion molecule precursor | P32736 | 84 | 2 | 1 | 0.75 | 2F, 2R |
| Protein kinase C and casein kinase substrate in neurons 1 | Q9Z0W5 | 147 | 4 | 1 | 0.84 | 1 |
| 28 kDa heat- and acid-stable phosphoprotein | Q62785 | 76.25 | 6 | 1 | 0.5 | 1, 2F, 2R |
| Pyruvate dehydrogenase E1 component alpha subunit | P26284 | 81.48 | 4 | 2 | 0.98 | 2F, 2R, 3F |
| Pyruvate dehydrogenase (Lipoamide) beta | Q6AY95 | 102 | 2 | 1 | 1.07 | 3R |
| PDZ domain-containing protein 2 | Q9QZR8 | 72.55 | 10 | 2 | 1.1 | 2F, 2R |
| 6-phosphofructokinase, liver type | P30835 | 94 | 5 | 1 | 0.71 | 3R |
| Prohibitin-2 | Q5XIH7 | 103 | 4 | 2 | 0.87 | 1, 2F, 3F |
| Phosphatidylinositol-4,5-bisphosphate 3-kinase catalytic subunit beta isoform | Q9Z1L0 | 62 | 6 | 1 | 0.74 | 3R |
| Phosphatidylinositol-4-phosphate 5-kinase type-2 alpha | Q9R0I8 | 54 | 5 |  |  | 1 |
| Myelin proteolipid protein (PLP) | P60203 | 108 | 4 | 1 | 1.02 | 2F, 3R |
| Outer dense fiber ODF3 | Q9Z221 | 57 | 8 |  |  | 2F |
| Serine/threonine-PP2B catalytic subunit alpha isoform | P63329 | 228 | 9 | 2 | 0.8 | 2F, 2R, 3F, 3R |
| Serine/threonine PP2B catalytic subunit beta isoform | P20651 | 94 | 4 | 1 | 0.91 | 3R |
| R3hdm_predicted protein | Q5PPF9 | 57 | 4 |  |  | 2F |
| RAF proto-oncogene serine/threonine-protein kinase | P11345 | 66.78 | 6 |  |  | 2R |
| Septin 10 | Q5PQK1 | 93 | 6 | 3 | 0.62 | 2F |
| G-septin beta | Q9WU35 | 60 | 6 | 1 | 0.93 | 3R |
| G-septin gamma | Q9R245 | 78 | 4 | 2 | 0.65 | 1, 2F, 2R |
| Septin-5 | Q9JJM9 | 84 | 4 | 1 | 0.83 | 1, 2R, 3R |
| Septin 7 | Q9WVC0 | 69 | 5 | 1 | 0.87 | 1, 2F |
| SH3P13S | O35104 | 82 | 5 | 2 | 1.12 | 2F |
| Solute carrier family 25 member 4 | Q05962 | 122.27 | 5 | 1 | 1.16 | 2F, 3F |
| Synaptosomal-associated protein 25 | P60881 | 127 | 5 | 1 | 0.64 | 1, 3R |
| Spna2 protein | Q6IRK8 | 121 | 12 | 5 | 0.86 | 1, 2F, 2R, 3R |
| Alpha II spectrin | P16086 | 182.47 | 11 | 4 | 0.9 | 3F |
| Signal transducer and activator of transcription 2 | Q5XI26 | 55 | 3 |  |  | 1 |
| Syntaxin-1B2 | P61265 | 148 | 8 | 2 | 0.92 | 1, 2F, 2R, 3F, 3R |
| Syntaxin-binding protein 1 | P61765 | 473 | 16 | 8 | 0.92 | 1, 2F, 2R, 3F, 3R |
| Synapsin-2 | Q63537 | 126 | 6 | 2 | 0.75 | 2F, 2R, 3F, 3R |
| Synaptotagmin I (First C2 Domain) | P21707 | 69 | 5 | 2 | 0.89 | 2F, 3F, 3R |
| Synaptotagmin-1 | P21707 | 117.49 | 3 | 2 | 0.76 | 3F, 3R |
| Synaptotagmin-2 | P29101 | 103.9 | 5 |  |  | 3F |
| Thy-1 membrane glycoprotein precursor | P01830 | 60 | 1 | 1 | 0.88 | 1 |
| Troponin I, cardiac muscle | P23693 | 56 | 4 |  |  | 1 |
| Tubulin, alpha 1 | P68370 | 239.36 | 8 | 3 | 0.71 | 2R, 3F |
| Tubulin, alpha 4 | Q5XIF6 | 209 | 9 | 1 | 1.02 | 1, 2F, 3F |
| Tubulin beta-2A chain | P04691 | 331 | 13 | 5 | 0.89 | 1 |
| Tubulin, beta 2c | Q6P9T8 | 260 | 11 | 4 | 0.9 | 1, 2F, 3F |
| Polyubiquitin | Q63429 | 64 | 3 |  |  | 1 |
| Ubiquinol-cytochrome c reductase core protein I | Q68FY0 | 293 | 9 | 3 | 0.91 | 1, 2F, 2R, 3F, 3R |
| Ubiquinol-cytochrome c reductase core protein II | Q5XIR3 | 227.87 | 10 | 5 | 0.92 | 1, 2F, 2R, 3F, 3R |
| Ubiquitin specific protease 52 | Q6IE70 | 56 | 4 |  |  | 1 |
| Rattus norvegicus utrophin | O55147 | 56 | 9 |  |  | 2F |
| Voltage-dependent anion-selective channel protein 1 | Q9Z2L0 | 364 | 14 | 6 | 0.91 | 1, 2F, 3F, 3R |
| Voltage-dependent anion-selective channel protein 2 | P81155 | 216 | 6 | 3 | 0.74 | 2F, 2R, 3F, 3R |
| Voltage-dependent anion-selective channel protein 3 | Q9R1Z0 | 145 | 6 | 1 | 0.67 | 2F, 2R, 3F, 3R |
| 14-3-3 protein beta/alpha | P35213 | 123 | 4 | 2 | 0.95 | 2F |
| 14-3-3 protein epsilon | P62260 | 142 | 7 | 2 | 0.87 | 2F, 3F, 3R |
| 14-3-3 protein gamma | P61983 | 137.63 | 7 | 3 | 1.02 | 1, 2F, 2R, 3F |
| 14-3-3 protein eta | P68511 | 115.31 | 7 | 2 | 0.83 | 3F, 3R |
| 14-3-3 protein theta | P68255 | 107.4 | 4 | 3 | 0.44 | 2R |
| 14-3-3 protein zeta/delta | P63102 | 159 | 6 | 3 | 0.68 | 1, 2R, 3F, 3R |
| alpha-2-globin chain |  | 87.81 | 4 | 1 | 0.88 | 2R |
| Beta 1 globin | Q9QUT6 | 238 | 7 | 4 | 0.96 | 2F, 3R |
| Chain A, Complex Of Alf4-Activated Gi-Alpha-1 With Rgs4 |  | 105 | 4 | 1 | 0.79 | 3R |
| Chain A, G Protein Heterotrimer Mutant Gi_alpha_1(G203a) Beta_1 Gamma_2 With Gdp Bound |  | 99.18 | 7 | 1 | 0.84 | 2R, 3F |
| Chain A, Rat Brain Hexokinase Type I Complex With Glucose And Inhibitor Glucose-6-Phosphate |  | 628.87 | 16 | 9 | 0.74 | 1, 2R, 3R |
| Chain A, N-Terminal Domain Of Syntaxin 1a |  | 111 | 4 | 1 | 0.92 | 3F, 3R |
| Chain B, Rat Brain Hexokinase Type I Complex |  | 522.5 | 15 | 6 | 0.93 | 2F, 3F |
| Chain B, Rat Liver F1-Atpase |  | 220 | 7 | 2 | 0.73 | 1 |
| Dynamin IIIbb isoform | Q9QXL9 | 134 | 7 | 3 | 1.35 | 1, 2F |
| EG3-1RVC | Q6XUZ6 | 60 | 3 |  |  | 1, 3R |
| Endoglin | Q6Q3E8 | 51 | 4 |  |  | 2F |
| Erythroid spectrin alpha | Q6XDA1 | 74.13 | 4 | 3 | 0.84 | 2R |
| Glutamate dehydrogenase (Fragment) | Q9T2P2 | 80 | 2 | 2 | 0.88 | 3R |
| GTP-binding regulatory protein Gs alpha-XL chain | P63095 | 112.73 | 8 | 2 | 1.04 | 1, 2R |
| Hemoglobin alpha-2 chain |  | 156 | 6 | 2 | 0.75 | 2F |
| Hemoglobin subunit beta-2 | P11517 | 183.89 | 7 | 3 | 0.86 | 2R, 3F |
| Histone H1D subtype | Q9QVB4 | 91.34 | 8 | 1 | 0.81 | 2R, 3F |
| Hypothetical protein (Fragment) | Q5M944 | 335.8 | 17 | 3 | 0.49 | 2R |
| LRRGT00016 | Q6TXI3 | 55 | 4 |  |  | 2F |
| Mutant type II hexokinase | O54892 | 107 | 6 | 1 | 0.65 | 3R |
| Neural cell adhesion molecule (version 1) |  | 67 | 1 | 1 | 1.52 | 2F |
| Plectin 5 | Q6S3A1 | 74 | 17 |  |  | 2F |
| Rat cytochrome C oxidase subunit VIC processed pseudogene, complete CDS |  | 68 | 2 | 1 | 0.7 | 2F, 3R |
| Similar to Tubulin alpha-2 chain (Alpha-tubulin 2) (predicted) |  | 264 | 11 | 2 | 1.24 | 1, 2F |
| Tubulin beta chain 15 |  | 360 | 13 | 6 | 0.78 | 1, 3F |
| Ubiquitin - bovine |  | 77 | 2 | 2 | 1.12 | 1, 2F |
| Ubiquitin / ribosomal protein L40, cytosolic |  | 64 | 2 |  |  | 3R |
